# Circulating and Tissue MicroRNA Profiles Associated with Pathological Complete Response in HER2+ Breast Cancer: A Systematic Review

**DOI:** 10.3390/ijms27167345

**Published:** 2026-08-17

**Authors:** Luis Bouz Mkabaah, Darragh T. McGovern, Eoin P. Kerin, Thomas O. Butler, Vinitha Richard, Aoife J. Lowery, Michael J. Kerin

**Affiliations:** Department of Surgery, The Lambe Institute for Translational Research, University of Galway, H91 YR71 Galway, Irelande.kerin3@universityofgalway.ie (E.P.K.); t.butler7@universityofgalway.ie (T.O.B.);

**Keywords:** HER2-positive breast cancer, MicroRNA, pathological complete response, neoadjuvant therapy, biomarkers

## Abstract

MicroRNAs (miRNAs) have emerged as promising biomarkers for treatment response prediction in breast cancer. However, the role of circulating and tumour-derived miRNAs in predicting pathological complete response (pCR) following neoadjuvant therapy in HER2-positive (HER2+) breast cancer remains incompletely defined. To systematically evaluate the association between miRNA expression profiles and pCR in HER2+ breast cancer undergoing neoadjuvant systemic therapy, including chemotherapy with HER2-targeted agents. A systematic review of PUBMED, EMBASE, SCOPUS, and WEB OF SCIENCE databases was performed according to PRISMA guidelines. Studies evaluating circulating or tumour-derived miRNAs associated with pCR following neoadjuvant therapy in HER2+ breast cancer were included. Methodological quality was assessed using the QUIPS tool. Nine studies involving 937 HER2+ breast cancer patients were included. Overall, 39 unique miRNAs and four circulating miRNA signatures were associated with pCR outcomes. Seven studies evaluated circulating miRNAs, while two assessed tumour-derived miRNAs. The increased expression of six circulating miRNAs, four circulating signatures, and seventeen tumour-derived miRNAs was associated with pCR. Conversely, the increased expression of eleven circulating miRNAs and five tumour-derived miRNAs was associated with residual disease and non-pCR. MiR-210 was the only miRNA associated with response across more than one study, with an increased expression associated with residual disease in both circulating and tumour-derived analyses. Several circulating and tumour-derived miRNAs demonstrate potential as predictive biomarkers of pCR following HER2-targeted neoadjuvant therapy. However, substantial heterogeneity and limited validation currently restrict clinical implementation. Future prospective multicentre studies using standardised methodologies are required to clarify the role of miRNA profiling in personalised treatment strategies for HER2+ breast cancer.

## 1. Introduction

Breast cancer remains the most common malignancy diagnosed in women worldwide. In Western populations, approximately one in eight women will be diagnosed with breast cancer over the course of their lifetime [1]. The amplification and/or overexpression of the human epidermal growth factor receptor 2 (HER2/neu) gene is observed in approximately 15–30% of breast cancer cases globally [2]. Traditionally, HER2-positive (HER2+) breast cancer has been widely linked with aggressive disease biology and poor prognosis. However, the introduction of targeted agents and immunotherapy has significantly improved outcomes for this subtype of breast cancer [3].

Neoadjuvant chemotherapy (NAC) has become a cornerstone in contemporary multidisciplinary treatment of breast cancer [4]. Pathological complete response (pCR) is defined as the absence of residual invasive disease in the resected breast and sampled regional lymph nodes (ypT0/Tis ypN0) and is widely used as an indicator of response to NAC [5]. The addition of targeted treatments to NAC, as demonstrated by major international trials, including NOAH, TECHNO, and GEPARQUINTO, has shown superior pCR outcomes when compared with chemotherapy alone in HER2+ breast cancer patients [6,7,8]. However, the capacity to predict which patients are most likely to achieve pCR remains limited, highlighting an unmet need for robust predictive biomarkers to guide personalised treatment strategies.

MicroRNAs are small non-coding RNAs implicated in the regulation of gene expression [9]. MiRNAs have emerged as promising biomarkers due to their role in post-transcriptional translation and their involvement in key oncogenic pathways [10]. Importantly, miRNAs are detectable in both tumour tissue and circulating biofluids, offering potential as minimally invasive biomarkers for treatment response prediction [11]. Emerging evidence has demonstrated that microRNA expression profiles are associated with response to neoadjuvant therapy in HER2-positive breast cancer. However, their specific role in predicting pCR remains incompletely defined [12]. Therefore, this systematic review aims to evaluate the role of circulating and tumour-derived miRNAs in predicting pCR in patients with HER2-positive breast cancer undergoing neoadjuvant therapy.

## 2. Methods

### 2.1. Literature Search

A formal systematic search was performed of the PUBMED, EMBASE, SCOPUS, and WEB OF SCIENCE databases in accordance to the Preferred Reporting Items for Systematic Review and Meta-Analyses (PRISMA) checklist [13]. An initial predefined search strategy was outlined by the senior authors (A.J.L and M.J.K). Two authors (L.B.M and D.M) conducted a search of the four databases to identify studies suitable for inclusion, the latest of which occurred in January 2026. The search results from each database were exported and collated manually in Microsoft Excel (version 16.88; Microsoft Corporation, Redmond, WA, USA). Duplicate records were manually identified and removed, and all subsequent title, abstract, and full-text screening was performed manually by the reviewers. Discrepancies in opinions were arbitrated by third and fourth authors (E.P.K and V.R). The search terms were as follows: (breast cancer), (breast neoplasm), (HER2), (ERBB2), (microRNA), (miRNA), (pathologic complete response), (pCR), and (response), which were all linked with the Boolean operator ‘AND’ and ‘OR’. Studies were combined from the four databases and duplicate studies were then manually removed. All studies had their titles screened initially and studies considered to be relevant had their abstracts and full texts reviewed. All authors were involved in developing a predefined review protocol, which was subsequently prospectively registered on the International Prospective Register of Systematic Reviews (PROSPERO: CRD420261358615).

### 2.2. Eligibility Criteria

Studies meeting the following predetermined inclusion criteria were considered for inclusion in this systematic review: (1) studies including patients with histologically confirmed HER2+ breast cancer receiving/being treated with neoadjuvant systemic therapy; (2) studies reporting treatment response outcomes, specifically including pCR; (3) studies demonstrating an association between microRNA (miRNA) expression and pCR, including comparisons based on differential expression; (4) studies assessing miRNA expression derived from circulating (e.g., plasma or serum) or tumour tissue samples; and (5) studies had to provide full-text manuscripts to warrant inclusion.

The exclusion criteria were as follows: (1) studies involving cell lines, animal models, or purely mechanistic research; (2) studies in which it was not possible to extract HER2-specific data from the overall study cohort; (3) studies not reporting treatment response outcomes, including pCR, or not linking miRNA expression to pCR; (4) studies not conducted in the neoadjuvant treatment setting; and (5) review articles, editorials, letters, conference abstracts without full text, and case reports.

### 2.3. Data Extraction and Statistical Analysis

Data were extracted from the included studies as to follow the predefined outcome measures. Clinicopathological characteristics were summarised as proportions using descriptive statistics, while miRNA expression profiles and treatment-related variables were reported descriptively in a narrative format. Substantial clinical and methodological heterogeneity precluded quantitative synthesis. Where reported, pCR was defined as ypT0/is ypN0. Minor variations in pCR definitions across studies were recorded during data extraction. The Quality in Prognosis Studies (QUIPS) tool was used to assess methodological quality and risk of bias across included prognostic biomarker studies [14].

## 3. Results

### 3.1. Literature Search

**Figure 1:**
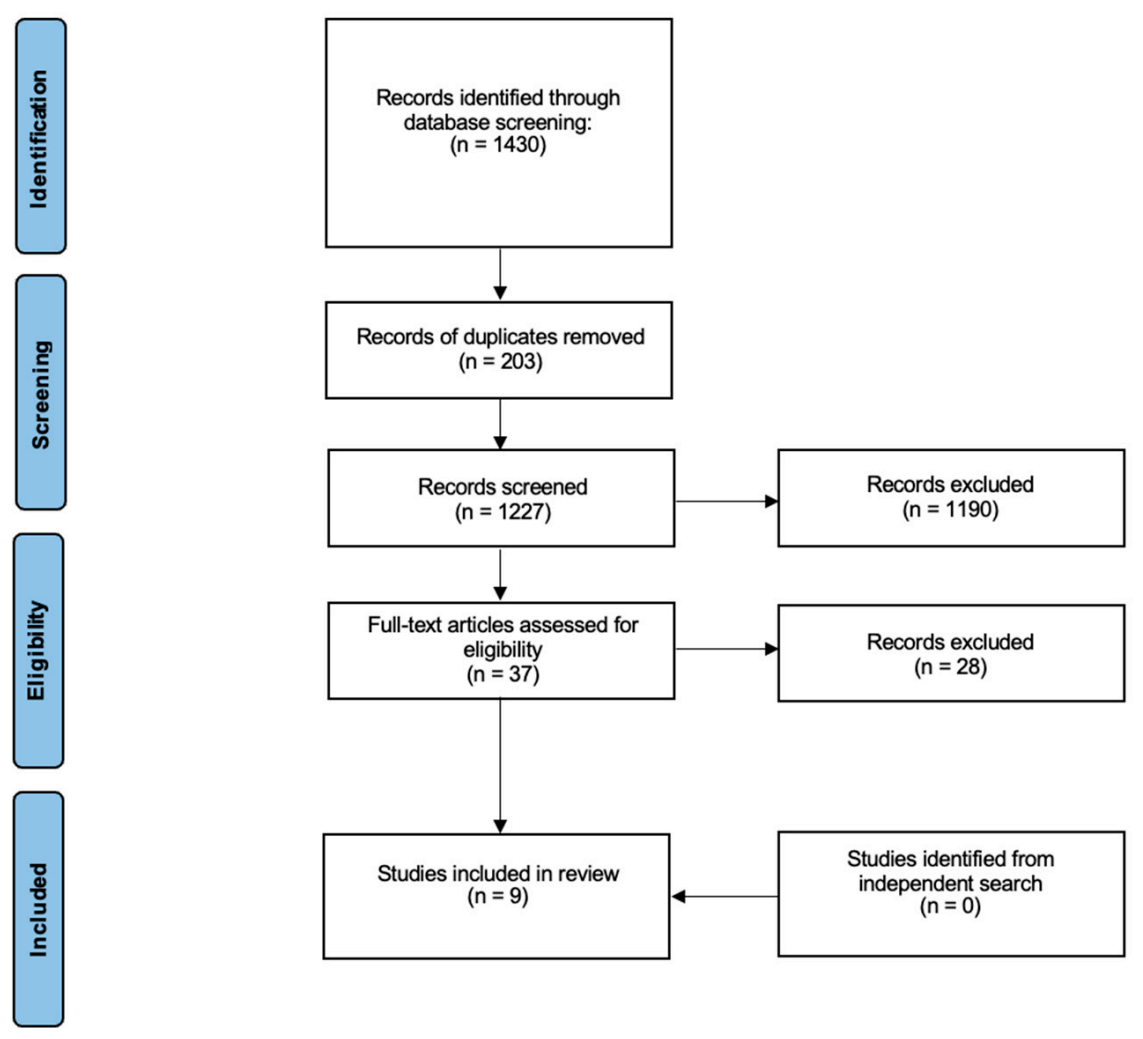
PRISMA flow diagram detailing the systematic search process.

The literature search yielded a total of 1430 studies. Following the removal of 203 duplicate studies, 1227 independent studies had their titles screened for relevance for inclusion in this study. Of these, 213 had their abstracts reviewed for relevance and 37 required full text manuscript review. Following full-text review, nine studies were included in this systematic review [15,16,17,18,19,20,21,22,23]. A PRISMA flow diagram detailing the systematic search process is outlined in Figure 1.

### 3.2. Study Characteristics and Clinicopathological Data

A total of 937 patients with HER2+ breast cancer across nine studies were included. Of the included studies, 55.6% (5/9) were prospective in design. The study characteristics are outlined in Table 1. The median age of the included patients was 50.0 years (range 21–83) (five studies). Where reported, 46.6% of patients were oestrogen receptor-positive (ER+) (268/575—four studies) and 48.9% were progesterone receptor-positive (PR+) (46/94—two studies). Tumour stage data demonstrated that 63.6% of cases were T1–T2 disease (500/786—five studies), while 36.2% were T3–T4 (284/784—three studies). Nodal staging showed that 89.5% of patients had N0–N1 disease (645/721—four studies), while 9.8% had N2 or higher nodal involvement (77/784—three studies). The basic clinicopathological parameters are summarised in Table 2.

Where diagnostic criteria were explicitly reported, HER2+ disease was defined as IHC 3+ or HER2 gene amplification by FISH in equivocal/IHC 2+ cases, in accordance with contemporaneous ASCO/CAP criteria [24]. Trial-based studies used the HER2 eligibility criteria of their respective parent trials. Ki-67 was variably assessed as a clinicopathological marker but was not used to define HER2 positivity.

### 3.3. Quality Assessment of Included Studies

Quality assessment using the QUIPS tool demonstrated generally moderate methodological quality across the included studies. Risk of bias was consistently low for pCR outcome measurement, as pathological response was assessed using standardised histopathological criteria across all studies. The principal sources of bias are related to study confounding, statistical analysis, and miRNA measurement methodology, particularly in smaller exploratory studies lacking external validation cohorts or predefined expression thresholds. Quality assessment of included studies is highlighted in Table 3.

### 3.4. MiRNA Expression and pCR

The nine studies included in this review identified a total of 39 individual miRNAs and four multivariate circulating miRNA signatures whose expression was associated with pCR status, including both pCR and non-pCR outcomes, following neoadjuvant therapy in HER2+ breast cancer. Circulating miRNAs were defined as miRNAs measured in blood-derived samples, including serum, plasma, whole blood, and plasma-derived exosomes, while circulating miRNA signatures referred to combinations of multiple circulating miRNAs evaluated together as predictive models. The results from seven studies assessed circulating miRNAs (77.8%), while two studies evaluated tumour-derived miRNAs (22.2%). The increased expression of six circulating miRNAs, four circulating miRNA signatures, and seventeen tumour-derived miRNAs was associated with achieving pCR. In contrast, the increased expression of eleven circulating miRNAs and five tumour-derived miRNAs was associated with residual disease and non-pCR. Notably, the overexpression of miR-210 was consistently associated with non-pCR across both circulating and tumour-based analyses in two studies [16,18].

### 3.5. Clinicopathological Associations

Clinicopathological associations were reported in a limited number of studies. Stevic et al. identified associations between exosomal miRNA expression and several clinicopathological parameters within the HER2-positive subgroup, including an association between miR-660 and nodal status (*p* = 0.016) [19]. Zhang et al. reported an association between serum miR-222-3p expression and presenting nodal status (*p* = 0.016), while no significant associations were observed with age, tumour stage, ER status, PR status, or Ki-67. An interaction between miR-222-3p expression and Ki-67 was also observed in relation to pCR (*p* = 0.025) [22].

### 3.6. MiRNA Expression from Tumour Tissue

Overall, 92 patients provided solid tumour tissue samples for evaluation (9.8%) (two studies) [17,18]. In total, 22 unique tumour-derived miRNAs were identified. Of these, a relatively higher expression of 17 miRNAs was associated with achieving pCR, while an increased expression of five miRNAs was associated with residual disease and non-pCR. de Mattos-Arruda et al. reported that elevated tumour miR-21 expression was associated with resistance to neoadjuvant trastuzumab chemotherapy and a reduced likelihood of pCR. Ohzawa et al. identified 21 differentially expressed tumour miRNAs, of which 17 were relatively enriched in patients achieving pCR, while increased expression of miR-210, miR-31-3p, miR-449a, and miR-449b-5p was associated with non-pCR following neoadjuvant trastuzumab-based chemotherapy. Tumour-derived miRNA profiles are summarised in Table 4, with corresponding fold-change values and measures of statistical significance, where reported, provided in Supplementary Table S1.

### 3.7. MiRNA Expression from Liquid Biopsy

Overall, 845 HER2+ patients provided liquid biopsy (i.e., blood, serum, or plasma) for the evaluation of ct-miRNA (90.2%), as provided by seven studies [15,16,19,20,21,22,23]. A total of 14 unique circulating miRNAs were identified, alongside four multivariate circulating miRNA signatures. Of these, increased expression of six circulating miRNAs and all four circulating miRNA signatures was associated with achieving pCR. In contrast, 11 circulating miRNAs were associated with residual disease and non-pCR. Wu et al. reported that higher serum levels of miR-375, miR-184, miR-1299, and miR-196a were associated with pCR, while increased levels of miR-381, miR-410, and miR-1246 were associated with non-pCR. Jung et al. demonstrated that elevated baseline plasma miR-210 expression was associated with residual disease following neoadjuvant trastuzumab-based chemotherapy. Stevic et al. identified lower levels of exosomal miRNAs, including miR-155, miR-301, miR-27b, miR-193b, and miR-660, were associated with increased likelihood of achieving pCR. Di Cosimo et al. derived four multivariate circulating miRNA signatures associated with pCR, which were validated in an independent cohort. Additional circulating biomarkers, including miR-148a-3p and miR-374a-5p, demonstrated increased expression during treatment in patients achieving pCR, while a low baseline expression of miR-222-3p and reduced mid-treatment expression of miR-145 were also associated with improved response. Detailed circulating miRNA profiles are summarised in Table 5. The functional roles of included miRNAs and gene targets are outlined in Table 6. Effect estimates and measures of statistical significance, where reported, are provided in Supplementary Table S2.

### 3.8. Timepoints of Sample Collection

All included studies reported timing of sample collection. Pre-treatment sampling prior to initiation of NAC was the most frequently reported timepoint and was performed in all nine studies. Six studies obtained samples exclusively prior to treatment initiation, including serum, plasma, tumour core biopsy, or whole blood specimens. Two studies incorporated longitudinal sampling designs, with repeat sampling performed during treatment; Jung et al. obtained plasma samples pre-NAC and two weeks following surgery, while both Di Cosimo et al. studies collected plasma samples at baseline and again two weeks after initiation of systemic therapy. Davey et al. additionally evaluated circulating miRNAs at both pre-treatment and mid-treatment timepoints during trastuzumab-based NAC.

**Figure 2:**
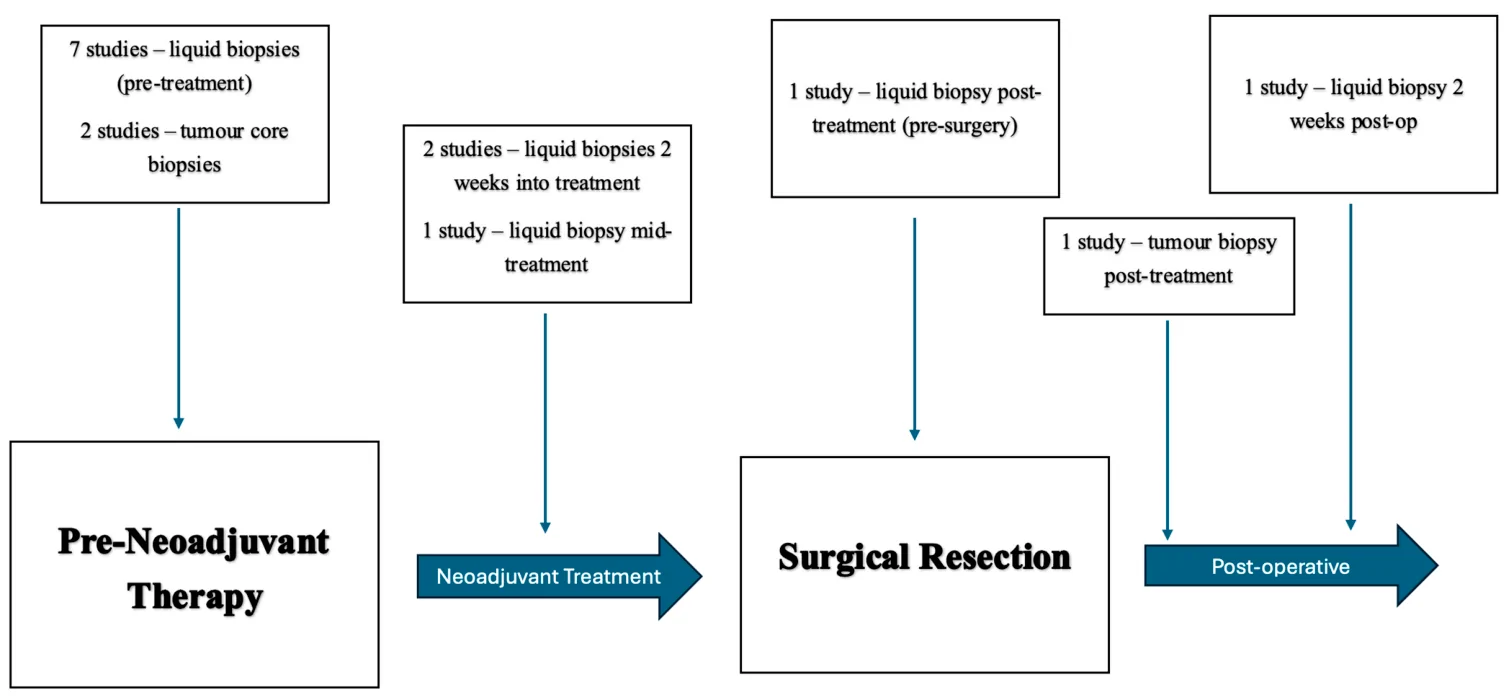
Timepoints at which liquid and solid tumour biopsies were collected across the included studies. Pre-treatment liquid biopsies were collected in seven studies [15,16,19,20,21,22,23], while pre-treatment tumour core biopsies were collected in two studies [17,18]. Liquid biopsies were collected two weeks after treatment initiation in two studies [20,21] and at mid-treatment in one study [23]. One study collected a liquid biopsy following completion of neoadjuvant treatment but prior to surgery [19], one collected tumour tissue following treatment [17], and one collected a liquid biopsy two weeks post-operatively [16].

Tumour-derived miRNA analyses were performed using pre-treatment core biopsy specimens, with one study by De Mattos-Arruda et al. also including paired post-treatment samples in a subset of patients. Timepoints at which liquid and solid tumour biopsies were obtained are illustrated in Figure 2.

## 4. Discussion

To our knowledge, this is the first systematic review specifically evaluating circulating and tumour-derived miRNAs associated with pCR following neoadjuvant therapy in HER2-positive breast cancer. This work successfully identified 39 unique individual miRNAs and four multivariate circulating miRNA signatures as being associated with pCR following neoadjuvant therapy in HER2+ breast cancer. These findings build upon previous translational research evaluating the broader role of circulating and tumour-derived miRNAs in predicting response to neoadjuvant and HER2-targeted therapies, as identified in a systematic review by Davey et al. However, despite the promising translational potential of these biomarkers, substantial methodological heterogeneity across studies currently limits their immediate clinical applicability.

The predominance of studies evaluating circulating miRNA identified in this review reflects the growing clinical interest in liquid biopsy as a minimally invasive tool for translational research in the field of breast cancer. Compared with tumour tissue sampling, circulating biomarkers obtained from serum, plasma, or whole blood offer several advantages, including reduced procedural burden, the ability to perform serial sampling, and the potential to capture and monitor dynamic changes during NAC [35]. Notably, the majority of included studies focused on baseline pre-treatment sampling, suggesting that the current research efforts remain largely directed toward identifying predictive biomarkers prior to treatment initiation. However, several studies, including those by Di Cosimo et al. and Davey et al., also demonstrated that dynamic changes in circulating miRNA expression during treatment may be associated with pCR, highlighting the importance of longitudinal biomarker assessment [20,21,23]. Notably, early changes in circulating miRNAs were demonstrated by Di Cosimo et al., while Davey et al. identified mid-treatment changes in miR-145 associated with pCR, and Jung et al. demonstrated an association between circulating miR-210, trastuzumab sensitivity and tumour presence [16,20,21,23]. These approaches are particularly relevant in HER2+ breast cancer, where the early identification of treatment-sensitive or treatment-resistant tumours could help guide more personalised treatment strategies, including treatment escalation or de-escalation where appropriate.

Only one individual miRNA, miR-210, was identified across more than one included study. Notably, increased miR-210 expression was associated with residual disease in both circulating and tumour-derived analyses, supporting its potential biological relevance as a marker of treatment resistance. The limited overlap of other miRNAs reduces confidence in their reproducibility, although this may partly reflect substantial methodological heterogeneity between studies. MiR-210 has previously been implicated in hypoxia-related tumour pathways and treatment resistance, supporting its potential biological relevance in HER2+ breast cancer [16]. MiR-210 is a key hypoxia-responsive miRNA regulated by HIF-1α and has been implicated in tumour survival, angiogenesis, metabolic adaptation, and treatment resistance. Emerging preclinical evidence suggests that direct inhibition of miR-210 biogenesis can reverse hypoxia-driven adaptive responses in breast cancer cells, highlighting its potential as both a predictive biomarker and therapeutic target [36]. Similarly, elevated tumour expression of miR-21 was associated with a reduced likelihood of achieving pCR, consistent with its recognised oncogenic role in breast cancer progression and therapeutic resistance [17]. MiR-21 promotes tumour progression through the suppression of tumour suppressor genes such as PTEN and PDCD4, leading to the activation of pro-survival signalling pathways. More recently, miR-21 has also attracted interest as a potential therapeutic target for overcoming treatment resistance [37,38]. While miR-21 and miR-210 were among the most extensively discussed biomarkers, several additional miRNAs identified in this review have previously been linked with breast cancer biology. For example, miR-155, miR-222-3p, miR-301, and miR-301b have been associated with tumour progression, treatment resistance, and HER2 signalling, while miR-148a-3p, miR-184, and miR-193b have been reported to exhibit tumour suppressive functions in breast and other solid malignancies [38,39,40,41,42]. Interestingly, a number of these miRNAs demonstrated associations with pCR that were not always consistent with their traditionally described biological roles, highlighting the context-dependent nature of miRNA function in HER2-positive breast cancer. Accordingly, the functional classifications presented in Table 6 reflect previously reported roles in the literature and should be interpreted as context-dependent rather than absolute classifications. Furthermore, the predictive value observed in multivariate miRNA signatures from NeoALTTO suggests that miRNA expression profiles may provide greater clinical utility than individual miRNAs alone [20].

Despite these promising findings, several important methodological limitations pose challenges in the translational utility of miRNAs in HER2+ breast cancer. Considerable heterogeneity was observed across the included studies with respect to the tissue source, timing of sample acquisition, treatment regimens, and analytical methodology. Studies evaluated miRNAs derived from serum, plasma, whole blood, and tumour tissue samples, while sampling timepoints ranged from baseline pre-treatment assessment to mid-treatment and post-treatment analysis. Furthermore, variability in miRNA extraction techniques, normalisation strategies, and expression thresholds may significantly influence reproducibility between studies [43,44]. Most studies also included relatively small patient cohorts and lacked external validation, limiting the generalisability of findings. Differences between circulating and tumour-derived miRNA profiles, along with dynamic alterations in expression during treatment, may further complicate interpretation. Clinicopathological associations were also inconsistently evaluated across studies, limiting the assessment of whether miRNA expression is independently related to established clinical and pathological predictors of treatment response. Long-term oncological outcomes were also infrequently reported, with Zhang et al. being the only included study to demonstrate that a pCR-associated miRNA, miR-222-3p, was also associated with DFS and OS [22]. Longer-term follow-up is therefore required to determine whether response-associated miRNAs also have prognostic value.

Another challenge in interpreting miRNA biomarkers is that their expression and biological role may vary depending on the clinical and molecular context. Several miRNAs identified in this review demonstrated associations with pCR that may appear inconsistent with their traditionally described biological roles in the literature. For example, certain miRNAs, such as miR-193b and miR-381, are characterised as tumour suppressor miRNAs in the literature [28,32]. However, they were associated with residual disease or non-pCR within HER2+ cohorts included in this review. These findings reflect the complex and dynamic nature of miRNA regulation in breast cancer biology [45]. Importantly, the established functional roles of miRNAs are derived from studies evaluating heterogeneous breast cancer populations rather than HER2-specific disease [46]. Given the unique molecular signalling pathways associated with HER2 disease, the biological behaviour of specific miRNAs may differ substantially within this subtype. An additional challenge is that circulating miRNAs may not exclusively reflect tumour biology. Their expression may also be influenced by host-specific factors, including immune responses, inflammation, and treatment-related systemic effects, which may contribute to variability between studies. Circulating miRNAs may reflect ongoing tumour cell turnover, treatment-induced cell death, or active release from tumour cells, potentially providing a dynamic measure of treatment response that cannot be captured by static tissue biomarkers alone.

A notable finding of this review is the absence of recent studies specifically evaluating miRNAs as predictors of pathological complete response (pCR) in HER2+ breast cancer. Although several studies published since 2022 have continued to investigate miRNAs in HER2+ disease, their focus has largely shifted towards treatment resistance, prognosis, and broader measures of therapeutic response rather than pCR itself. For example, Rezaei et al. identified altered plasma expression of miR-195-5p, miR-34c-3p, and miR-1246 in trastuzumab-resistant HER2+ breast cancer patients, while Di Cosimo et al. reported a three-miRNA prognostic signature comprising miR-185-5p, miR-146a-5p, and miR-22-3p that was associated with event-free survival following trastuzumab-based neoadjuvant therapy [47]. More recently, Peng et al. demonstrated an association between serum miR-1 expression and pCR following NAC; however, this analysis was performed in a mixed breast cancer cohort and was not specific to HER2+ disease [48]. Consequently, no additional studies meeting the predefined eligibility criteria were identified despite continued interest in miRNA biomarkers. This highlights an important translational gap in the literature and suggests that progress towards clinically applicable miRNA biomarkers for pCR prediction in HER2+ breast cancer has remained limited. This may reflect persistent challenges in standardisation, reproducibility, threshold definition, and external validation, which are well-recognised barriers in circulating miRNA biomarker research [49].

In conclusion, this systematic review demonstrates that several circulating and tumour-derived miRNAs are associated with pCR following HER2-targeted NAC. Circulating miRNAs, particularly those evaluated through longitudinal liquid biopsy approaches, represent promising minimally invasive biomarkers for treatment response prediction in HER2+ breast cancer. Nevertheless, substantial methodological heterogeneity and limited prospective validation pose a challenge in the clinical implementation of miRNAs. Future studies should also prioritise the validation of recurrent miRNA candidates across independent cohorts and biological compartments, as the limited overlap observed between existing studies currently restricts assessment of reproducibility and clinical utility. Furthermore, future multicentre prospective studies incorporating standardised methodologies and dynamic biomarker assessment are required to clarify the role of miRNA profiling in personalised treatment strategies for HER2+ breast cancer. Ultimately, the clinical value of miRNA profiling may lie in its ability to distinguish responders from non-responders prior to treatment initiation, enabling more personalised therapeutic strategies and reducing unnecessary treatment-related toxicity. Studies may need to move beyond isolated miRNA biomarkers and evaluate integrated predictive models incorporating miRNA profiles with other emerging biomarkers, such as ctDNA, tumour-infiltrating lymphocytes, radiomics, and clinicopathological factors.

## Figures and Tables

**Table 1:** Included studies assessing miRNA expression and their role in predicting pathological complete response [15,16,17,18,19,20,21,22,23].

Author	Year	Country	Tissue	Total N	HER2+ N (Analysed)	LOE	Pathology Confirmation	Treatment	Timing of Sampling	Technique	miRNA Selection	Validation
Wu	2012	US	Serum	42	23	Prospective	Yes	Doxorubicin/cyclophosphamide and Trastuzumab followed by carboplatin and nab-paclitaxel	Pre-NAC (baseline)	qRT-PCR	Microarray	Yes
Jung	2012	US	Plasma	29	29	Prospective	Yes	Fluorouracil, Epirubicin, Cyclophosphamide, and Trastuzumab	Pre-NAC and 2 weeks post-op	qRT-PCR	Selected from historical laboratory findings	No
de Mattos-Arruda	2015	Italy/Spain	Tumour (FFPE)	73	52	Retrospective	Yes	Anthracycline, Docetaxel, and Trastuzumab	Pre-treatment core biopsy ± paired post-treatment (subset)	qRT-PCR	Selected from the literature	Yes
Ohzawa	2017	Japan	Tumour (FFPE)	47	40	Retrospective	Yes	Trastuzumab-based NAC	Pre-treatment core biopsy	qRT-PCR	miRNA array	No
Stevic	2018	Germany	Plasma	435	211	Prospective (GeparSixto Trial)	Yes	Paclitaxel + doxorubicin ± carboplatin + trastuzumab + lapatinib	Pre-treatment ± post-treatment	Western Blot/ELISA	miRNA array	No
Di Cosimo	2019	Italy	Plasma	429	429	Prospective (NeoALLTO trial)	Yes	Trastuzumab, lapatinib, and paclitaxel	Pre-treatment + 2 weeks into treatment	qRT-PCR	miRNA array	Yes
Di Cosimo	2020	Italy	Plasma	52	52	Retrospective	Yes	Trastuzumab, lapatinib, and paclitaxel	Pre-treatment + 2 weeks into treatment	qRT-PCR	miRNA array	Yes
Zhang	2020	China	Serum	65	65	Retrospective	Yes	Paclitaxel, Cisplatin, and Trastuzumab	Pre-treatment	qRT-PCR	Selected from historical laboratory findings	No
Davey	2022	Ireland	Blood	120	36	Prospective	Yes	Trastuzumab-based NAC	Pre-treatment and mid-treatment	qRT-PCR	Selected from the literature	No

Abbreviations: ELISA, enzyme-linked immunosorbent assay; HER2+, Human epidermal growth factor receptor-2 positive; miRNA, microRNA; N, number; NAC, neoadjuvant chemotherapy; LOE, level of evidence; qRT-PCR, quantitative real-time polymerase chain reaction; US, United States.

**Table 2:** Basic clinicopathological parameters from the included studies.

Author	Median Age	Over 50	Age Range	pCR Achieved (% of Total N)	ER+	PR+	T1	T2	T3	T4	N0	N1	N2	N3
Wu	NR	NR	NR	12 (52.2%)	NR	NR	NR	NR	NR	NR	NR	NR	NR	NR
Jung	52	NR	21–70	18 (62.1%)	13	8	3	20	6	0	10	19	0	0
de Mattos-Arruda	NR	NR	NR	28 (53.8%)	NR	NR	NR	NR	NR	NR	NR	NR	NR	NR
Ohzawa	55	NR	31–83	15 (37.5%)	NR	NR	NR	NR	NR	NR	NR	NR	NR	NR
Stevic	47	91 (43.1%)	21–78	113 (53.6%)	NR	NR	167		42		106	103	0	NR
Di Cosimo	50	NR	23–80	150 (35%)	201/429 (46.9%)	NR	261 (60.8%)		168/429 (39.2%)		361/429 (84.1%)		68/429 (15.9%)	0
Di Cosimo	48.5	NR	25–70	16 (31%)	26 (50%)	NR	33/52 (63%)		19/52 (36%)		43/52 (83%)		9/52 (17%)	0
Zhang	NR	39	NR	31 (47.7%)	28	38	2	14	28	21	9	49	2	5
Davey	NR	NR	NR	15 (41.7%	NR	NR	NR	NR	NR	NR	NR	NR	NR	NR

Abbreviations: ER+, estrogen receptor-positive; N0, no nodal involvement; N, Number; N1, nodal status stage 1; N2, nodal status stage 2; N3, nodal status stage 3; NR, not reported; pCR, pathological complete response; PR+, progesterone receptor-positive; T1, tumour stage 1; T2, tumour stage 2; T3, tumour stage 3; T4, tumour stage 4.

**Table 3:** Quality in Prognosis Studies (QUIPS) tool used to assess the quality of the included studies.

Study	Study Participation	Study Attrition	miRNA Measurement	pCR Outcome Measurement	Study Confounding	Statistical Analysis/Reporting	Overall Risk
Wu	Moderate	Moderate	Moderate	Low	High	Moderate	Moderate
Jung	Low	Moderate	Moderate	Low	Moderate	Moderate	Moderate
de Mattos-Arruda	Moderate	Moderate	Low	Low	Moderate	Moderate	Moderate
Ohzawa	Moderate	Moderate	Moderate	Low	High	High	High
Stevic	Low	Low	Moderate	Low	Moderate	Moderate	Moderate
Di Cosimo	Low	Low	Low	Low	Low	Low	Low
Di Cosimo	Low	Moderate	Low	Low	Moderate	Moderate	Moderate
Zhang	Moderate	Moderate	Moderate	Low	Moderate	Moderate	Moderate
Davey	Low	Low	Low	Low	Moderate	Moderate	Moderate

**Table 4:** MicroRNAs expression patterns in tumour [17,18].

Author	Year	miRNA	Clinical Association
de Mattos-Arruda et al.	2015	miR-21	Elevated tumour miR-21 expression associated with resistance to neoadjuvant trastuzumab chemotherapy and decreased likelihood of achieving pCR
Ohzawa et al.	2017	miR-106b-3p, miR-1180, miR-1238-5p, miR-142-5p, miR-150-5p, miR-181c-5p, miR-182-5p, miR-200a-5p, miR-218-5p, miR-3609, miR-362-5p, miR-3620-3p, miR-4418, miR-4506, miR-4657, miR-505-3p, miR-505-5p	Reduced tumour expression associated with non-pCR; relatively higher expression associated with achieving pCR following neoadjuvant trastuzumab-based chemotherapy (differential expression analysis)
Ohzawa et al.	2017	miR-210, miR-31-3p, miR-449a, miR-449b-5p	Increased tumour expression associated with non-pCR following neoadjuvant trastuzumab-based chemotherapy (differential expression analysis)

**Table 5:** MicroRNAs expression patterns in liquid biopsy [15,16,19,20,21,22,23].

Author	Year	miRNA	Clinical Association
Wu et al.	2012	miR-375, miR-184, miR-1299, miR-196a	Higher circulating serum levels associated with pCR to neoadjuvant chemotherapy
Wu et al.	2012	miR-381, miR-410, miR-1246	Increased circulating serum levels associated with non-pCR
Jung et al.	2012	miR-210	Higher circulating plasma levels at baseline associated with residual disease following neoadjuvant trastuzumab-based chemotherapy
Stevic et al.	2018	miR-155, miR-301, miR-27b, miR-193b, miR-660	Higher exosomal levels associated with increased likelihood of pCR following neoadjuvant (logistic regression models)
Di Cosimo et al. (NeoALTTO)	2019	Four ct-miRNA signatures	Four multivariate circulating miRNA signatures independently predicted pCR following HER2-targeted neoadjuvant therapy (validated in independent cohort)
Di Cosimo et al.	2020	miR-148a-3p, miR-374a-5p	Increased circulating plasma levels during treatment (week 2 vs. baseline) associated with pCR following trastuzumab-based neoadjuvant therapy
Zhang et al.	2020	miR-222-3p	Low baseline serum expression associated with higher pCR rates; high expression associated with reduced pCR (trastuzumab resistance)
Davey et al.	2022	miR-145	Reduced circulating expression (mid-treatment vs. baseline) associated with achieving pCR

**Table 6:** Functional roles of included microRNAs and associated gene targets.

miRNA(s)	Role	Gene Target/Pathway	Reference
let-7a-3p	Tumour suppressor	RAB10	[18]
miR-21	Oncomir	PTEN, PDCD4	[17]
miR-27b	Oncomir	CYP1B1, PDHX	[25]
miR-31-3p	Chemoresistance-associated oncomir	RASA2, RHOA, ITGA5, RDX	[18]
miR-34b-5p	EMT regulator	ARHGAP1	[18]
miR-145	Oncomir	ERBB3, IGF1R, FSCN1	[23]
miR-148a-3p	Tumour suppressor	ERBB3, IGF1R, IRS1	[21]
miR-150-5p	Oncomir	SRCIN1	[18]
miR-155	Oncomir	FOXO3A, RUNX2	[26]
miR-184	Tumour suppressor	AKT2, GSK3A, PRAS40, CSF1	[27]
miR-193b	Tumour suppressor	PLAU, RAB22A	[28]
miR-196a	Oncomir	ANXA1, SPRED1	[29]
miR-210	Hypoxia-related oncomir	ISCU, MNT	[16,18]
miR-218-5p	Chemosensitivity regulator	SOST, SFRP2	[18]
miR-222-3p	Oncomir and trastuzumab resistance regulator	PTEN, FOXO1, SRC	[22]
miR-301	Oncomir	PTEN, FOXF2, BBC3, COL2A1	[30]
miR-301b	Oncomir	CYLD, NR3C2	[18]
miR-374a-5p	Aberrantly expressed oncomir	ARRB1	[21]
miR-375	Trastuzumab sensitivity regulation	IGF1R	[31]
miR-381	Tumour suppressor	CTNNB1, RHOA, ROCK1, MYC	[32]
miR-410	Anti-metastatic regulation	SNAI1	[33]
miR-429	Chemoresistance-associated oncomir	FN1	[18]
miR-449a	Tumour suppressor	FASN	[18]
miR-449b-5p	Tumour suppressor	FASN, RPRD1B	[18]
miR-1246	Oncomir and trastuzumab resistance	NFE2L3	[26]
miR-1299	Tumour suppressor	CCND1, CDK6, CDK8	[34]

## Data Availability

No new data were created or analyzed in this study. Data sharing is not applicable to this article.

## Supplementary Materials

The following supporting information can be downloaded at: https://www.mdpi.com/article/10.3390/ijms27167345/s1.
