# Supplementary material for: Circulating and Tissue MicroRNA Profiles Associated with Pathological Complete Response in HER2+ Breast Cancer: A Systematic Review
Source: Int J Mol Sci. 2026 Aug 17;27(16):7345. doi: 10.3390/ijms27167345 (PMC13513851; doi:10.3390/ijms27167345)
Supplement: Supplementary file 1 [file ijms-27-07345-s001.zip › ijms-4423062-supplementary.pdf]

Supplementary Tables

Circulating and Tissue MicroRNA Profiles Associated with Pathological Complete Response in HER2+ Breast Cancer: A Systematic Review

Supplementary Table S1. Detailed tumour-derived miRNAs associated with pathological response.

Reference study	miRNA / signature	Direction of expression	Fold change / effect estimate	Statistical significance	Association with pCR
De Mattos-Arruda et al. 2015 (17)	miR-21	Higher in residual disease / non-pCR	-4.989 (as reported)	p=0.030; validation cohort p=0.012	Higher tumour miR-21 associated with resistance to trastuzumab-chemotherapy and reduced likelihood of pCR
Ohzawa et al. 2017 (18)	miR-106b-3p	Higher in pCR	2.97	p=0.046	Higher tumour expression associated with pCR
Ohzawa et al. 2017 (18)	miR-1180	Higher in pCR	4.64	p=0.035	Higher tumour expression associated with pCR
Ohzawa et al. 2017 (18)	miR-1238-5p	Higher in pCR	3.00	p=0.046	Higher tumour expression associated with pCR
Ohzawa et al. 2017 (18)	miR-142-5p	Higher in pCR	2.73	p=0.035	Higher tumour expression associated with pCR
Ohzawa et al. 2017 (18)	miR-150-5p	Higher in pCR	2.05	p=0.030	Higher tumour expression associated with pCR
Ohzawa et al. 2017 (18)	miR-181c-5p	Higher in pCR	4.27	p=0.016	Higher tumour expression associated with pCR
Ohzawa et al. 2017 (18)	miR-182-5p	Higher in pCR	3.18	p=0.021	Higher tumour expression associated with pCR
Ohzawa et al. 2017 (18)	miR-200a-5p	Higher in pCR	4.85	p=0.028	Higher tumour expression associated with pCR
Ohzawa et al. 2017 (18)	miR-218-5p	Higher in pCR	5.99	p=0.013	Higher tumour expression associated with pCR
Ohzawa et al. 2017 (18)	miR-3609	Higher in pCR	3.84	p=0.026	Higher tumour expression associated with pCR
Ohzawa et al. 2017 (18)	miR-362-5p	Higher in pCR	3.33	p=0.049	Higher tumour expression associated with pCR
Ohzawa et al. 2017 (18)	miR-3620-3p	Higher in pCR	3.72	p=0.046	Higher tumour expression associated with pCR
Ohzawa et al. 2017 (18)	miR-4418	Higher in pCR	4.40	p=0.030	Higher tumour expression associated with pCR
Ohzawa et al. 2017 (18)	miR-4506	Higher in pCR	4.95	p=0.015	Higher tumour expression associated with pCR
Ohzawa et al. 2017 (18)	miR-4657	Higher in pCR	3.37	p=0.049	Higher tumour expression associated with pCR
Ohzawa et al. 2017 (18)	miR-505-3p	Higher in pCR	3.44	p=0.026	Higher tumour expression associated with pCR
Ohzawa et al. 2017 (18)	miR-505-5p	Higher in pCR	3.75	p=0.012	Higher tumour expression associated with pCR
Ohzawa et al. 2017 (18)	miR-210	Higher in non-pCR	2.22	p=0.046	Higher tumour expression associated with non-pCR
Ohzawa et al. 2017 (18)	miR-31-3p	Higher in non-pCR	2.04	p=0.043	Higher tumour expression associated with non-pCR
Ohzawa et al. 2017 (18)	miR-449a	Higher in non-pCR	6.44	p=0.028	Higher tumour expression associated with non-pCR
Ohzawa et al. 2017 (18)	miR-449b-5p	Higher in non-pCR	3.15	p=0.037	Higher tumour expression associated with non-pCR

Notes: Fold-change values and p values are reproduced as reported by the original studies. For De Mattos-Arruda et al., the negative fold-change value is retained exactly as reported; the direction of association is based on the authors' finding of higher miR-21 expression in residual disease. For Ohzawa et al., direction is presented according to the authors' reported regulation and accompanying description of pCR versus non-pCR. pCR, pathological complete response; RD, residual disease.

Supplementary Table S2. Detailed circulating miRNAs and circulating miRNA signatures associated with pathological response.

Reference study	miRNA / signature	Direction of expression	Fold change / effect estimate	Statistical significance	Association with pCR
Wu et al. 2012 (15)	miR-375	Higher in pCR	Log2 FC (pCR vs non-pCR): 3.02	p=1.10E-09	Higher serum expression associated with pCR
Wu et al. 2012 (15)	miR-184	Higher in pCR	Log2 FC (pCR vs non-pCR): 2.99	p=2.59E-09	Higher serum expression associated with pCR
Wu et al. 2012 (15)	miR-1299	Higher in pCR	Log2 FC (pCR vs non-pCR): 2.35	p=1.24E-06	Higher serum expression associated with pCR
Wu et al. 2012 (15)	miR-196a	Higher in pCR	Log2 FC (pCR vs non-pCR): 2.05	p=1.79E-05	Higher serum expression associated with pCR
Wu et al. 2012 (15)	miR-381	Higher in non-pCR	Log2 FC (pCR vs non-pCR): -1.69	p=2.48E-04	Higher serum expression associated with non-pCR
Wu et al. 2012 (15)	miR-410	Higher in non-pCR	Log2 FC (pCR vs non-pCR): -1.50	p=1.09E-03	Higher serum expression associated with non-pCR
Wu et al. 2012 (15)	miR-1246	Higher in non-pCR	Log2 FC (pCR vs non-pCR): -1.49	p=1.16E-03	Higher serum expression associated with non-pCR
Jung et al. 2012 (16)	miR-210	Higher at baseline in residual disease / non-pCR	Residual:pCR baseline expression ratio: 2.18	p=0.0359	Higher baseline plasma miR-210 associated with residual disease after trastuzumab-based therapy
Stevic et al. 2018 (19)	miR-27b	Higher baseline exosomal expression associated with pCR	Multivariable OR 1.28 (95% CI 1.00-1.63)	p=0.050	Predicted pCR in HER2-positive subgroup
Stevic et al. 2018 (19)	miR-155	Higher baseline exosomal expression associated with pCR	Multivariable OR 1.26 (95% CI 1.02-1.56)	p=0.035	Predicted pCR in HER2-positive subgroup
Stevic et al. 2018 (19)	miR-193b	Higher baseline exosomal expression associated with pCR	Multivariable OR 1.26 (95% CI 1.05-1.51)	p=0.012	Predicted pCR in HER2-positive subgroup
Stevic et al. 2018 (19)	miR-301	Higher baseline exosomal expression associated with pCR	Multivariable OR 1.32 (95% CI 1.07-1.64)	p=0.011	Predicted pCR in HER2-positive subgroup
Stevic et al. 2018 (19)	miR-660	Higher baseline exosomal expression associated with pCR	Multivariable OR 1.40 (95% CI 1.04-1.89)	p=0.027	Predicted pCR in HER2-positive subgroup
Di Cosimo et al. 2019 (20)	Lapatinib baseline ct-miRNA signature: miR-100-5p, miR-197-3p, miR-320c, miR-376c-3p, miR-874-3p	Multivariate circulating signature predictive of pCR	Component log2 FCs: 0.74, -0.71, 0.36, -1.95, 0.38; testing AUC 0.86 (95% CI 0.73-0.98)	Confirmed in independent testing set	Signature discriminated pCR from non-pCR
Di Cosimo et al. 2019 (20)	Lapatinib week-2 ct-miRNA signature: miR-100-5p, miR-144-3p, miR-362-3p	Multivariate circulating signature predictive of pCR	Component log2 FCs: 0.58, 0.66, 0.67; testing AUC 0.71 (95% CI 0.55-0.86)	Confirmed in independent testing set	Signature discriminated pCR from non-pCR
Di Cosimo et al. 2019 (20)	Trastuzumab week-2 ct-miRNA signature: miR-140-5p, miR-145-5p, miR-328-3p, miR-374a-5p, miR-574-3p	Multivariate circulating signature predictive of pCR	Component log2 FCs: 0.48, 0.26, -0.57, 0.69, 0.77; testing AUC 0.81 (95% CI 0.70-0.92)	Confirmed in independent testing set	Signature discriminated pCR from non-pCR
Di Cosimo et al. 2019 (20)	Lapatinib + trastuzumab week-2 ct-miRNA signature: miR-34a-5p, miR-98-5p, miR-100-5p	Multivariate circulating signature predictive of pCR	Component log2 FCs: -0.80, 0.51, -1.10; testing AUC 0.67 (95% CI 0.51-0.83)	Confirmed in independent testing set	Signature discriminated pCR from non-pCR
Di Cosimo et al. 2020 (21)	miR-148a-3p	Increased from baseline to week 2 in pCR	Median FC: 1.34 (pCR) vs 0.90 (non-pCR); adjusted OR 3.42 (95% CI 1.23-9.46)	Kruskal-Wallis p=0.008; multivariable p=0.018	Early increase during trastuzumab-based therapy associated with pCR
Di Cosimo et al. 2020 (21)	miR-374a-5p	Increased from baseline to week 2 in pCR	Median FC: 1.73 (pCR) vs 1.19 (non-pCR); adjusted OR 2.31 (95% CI 0.93-5.78)	Kruskal-Wallis p=0.048; multivariable p=0.073	Early increase during trastuzumab-based therapy associated with pCR in univariate analysis
Zhang et al. 2020 (22)	miR-222-3p	Lower baseline expression associated with pCR	Multivariable OR (high vs low) 0.258 (95% CI 0.070-0.958)	p=0.043	Low serum miR-222-3p group more likely to achieve pCR
Davey et al. 2022 (23)	miR-145	Decreased from baseline to mid-treatment in pCR	—	p=0.027	Reduced whole-blood miR-145 during NAC associated with pCR in HER2-positive disease

Notes: Fold change is shown where directly reported. Where fold change was not reported or was not applicable to a multivariate model, the original study's effect or performance estimate is shown instead. Stevic et al. odds ratios are from HER2-positive multivariable logistic regression models adjusted for age, nodal status, tumour size and grade. Di Cosimo et al. (2019) signature components are those retained in the final LASSO models; component log2 fold changes are from the training set and AUCs are from the independent testing set. A dash indicates that no fold-change/effect estimate was reported. AUC, area under the receiver operating characteristic curve; CI, confidence interval; ct-miRNA, circulating microRNA; FC, fold change; NAC, neoadjuvant chemotherapy; OR, odds ratio; pCR, pathological complete response.
